# Chirality of the biomolecules enhanced its stereospecific action of dihydromyricetin enantiomers

**DOI:** 10.1002/fsn3.1766

**Published:** 2020-07-28

**Authors:** Muhammad Umair, Saqib Jabbar, Tayyaba Sultana, Zubaria Ayub, Sheikheldin A. Abdelgader, Zhu Xiaoyu, Zhang Chong, Lu Fengxia, Bie Xiaomei, Lu Zhaoxin

**Affiliations:** ^1^ College of Food Science and Technology Nanjing Agriculture University Nanjing China; ^2^ Food Science Research Institute (FSRI) National Agricultural Research Centre (NARC) Islamabad Pakistan; ^3^ College of Public Administration Nanjing Agriculture University Nanjing China; ^4^ Institute of Home Sciences University of Agriculture Faisalabad Pakistan; ^5^ College of Animal Production Bahri University Khartoum Sudan

**Keywords:** cell integrity, dihydromyricetin, enantiopure, selective antimicrobial activity, stereo enantiomers, vine tea

## Abstract

The present study explores the effect of chirality of the biological macromolecules, its functional aspects, and its interaction with other food components. Dihydromyricetin (DHM) is a natural novel flavonol isolated from the vine tea (*Ampelopsis grossedentata)* leaves. However, limited progress in enantiopure separation methods of such compounds hinder in the development of enantiopure functional studies. This study is an attempt to develop a simple, accurate, and sensitive extraction method for the separation of the enantiopure DHM from vine tea leaves. In addition, the identification and purity of the extracted enantiopure (−)‐DHM were further determined by the proton nuclear magnetic resonance (^1^H‐NMR) and the carbon nuclear magnetic resonance (^13^C‐NMR). The study further evaluates the antimicrobial activity of isolated (‐)‐DHM in comparison with racemate (+)‐DHM, against selected foodborne pathogens, whereas the action mode of enantiopure (−)‐DHM to increase the integrity and permeability of the bacterial cell membrane was visualized by confocal laser scanning microscopy using green fluorescence nucleic acid dye (SYTO‐9) and propidium iodide (PI). Moreover, the morphological changes in the bacterial cell structure were observed through field emission scanning electron microscope. During analyzing the cell morphology of *B. cereus* (AS11846), it was confirmed that enantiopure (−)‐DHM could increase the cell permeability that leads to the released of internal cell constituents and, thus, causes cell death. Therefore, the present study provides an insight into the advancement of enantiopure isolation along with its antimicrobial effect which could be served as an effective approach of biosafety.

## Graphical abstract

The present study explores the effect of chirality of the biological macromolecules and its functional aspects along with the interaction with other major biological macromolecules. Dihydromyricetin (DHM) is a natural novel flavonol isolated from *Ampelopsis grossedentata*.

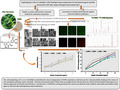

## 1 INTRODUCTION

The antibiotic abuse causes multi‐drug‐resistant (MDR) bacteria, which were critical necessitate to put into practice effective strategy substitutes for antibiotics. Several series of bactericidal strategies (nanomaterial, cationic, and anionic coating) have been opting to overcome its effect (Zhu, Dong, Chang, Li, & Wang, 2019). However, efficacy issues and irreversible biomolecules damage limit their application. Plants‐derived bioactive compound flavonoids demonstrate a diversity of biological and functional perspectives. These chiral flavonoids are attractive applicants for the development of bactericidal effects and drug discovery (Zhu et al., 2018). However, only very few of these flavanols are asserted as a resolved enantiomer.

Dihydromyricetin (DHM), extracted from vine tea (*Ampelopsis grossedentata*), has been widely used for promoting health benefits. The presence of two asymmetry substituted carbon atoms in the DHM chemical structure leads to the formulation of four possible stereoisomers (Wang, Xiong, Perumala, Fang, & Sun, 2016). The most common stereoisomer form of DHM is a racemate (±) DHM form rather than optically pure enantiomer, such as (+) DHM, and (−)‐DHM. Because any change in the pH value during the extraction or crystallization process, or prolonged high‐temperature exposure, or any direct in‐contact with the metal surface, may result in the racemization process that ultimately affects the enantioselective functional property (Park & Kim, 2016). So, it is imperative to develop a sensitive, reproducible, and semi‐preparative extraction method for the extraction of the desired enantiomer with the required level of enantiomeric excess from the racemic mixture. Thereby the successful extraction of stereoisomer form of DHM is used to understand the enantioselective functional property that will significantly advance with other flavanols asserted as a resolved enantiomer.

Chiral recognition and its interaction among other molecules is a significant step in natural biological processes. A recent development for enantiomers has highlighted the impact of specific stereochemistry of enantiomers and its configuration difference (due to repulsive forces between overlapping electron clouds) on the biological, metabolic, pharmacological, and toxicological activities in vitro and vivo (Lin et al., 2019; Zhou, Yu, & Zeng, 2014). It is also reported that enantiopure could play an essential role in drug discovery due to its effective biological compound coordination with receptor molecules, which can result in the desired effect with fewer side effects and more efficacious (Pinto et al., , 2020). Another study has been reported that the selective adhesion and differentiation of the enantiomer of cells and proteins (Qiu et al., 2018) as chiral arrangement plays a crucial function in biological activity. Therefore, the enantioselective functional behavior of enantiopure in comparison with the racemate mixture is imperative. But, the selective adsorption of proteins could induce cell differentiation, and selective toxicity to bacteria of chiral molecules which has not reported yet.

So far, a minimum number of studies have performed to assess the biological and functional aspects of optically pure (+) DHM and (−) DHM in comparison with racemate mixture form (+)‐DHM. In this, we have developed a simple, accurate, and sensitive semi‐preparative HPLC extraction method for the isolation, separation and purification of enantiomer, such as (−)‐DHM enantiomer and evaluated its antimicrobial capacity against selected foodborne pathogens. Further, the identity and the purity of the isolated enantiomer will be determined by proton nuclear magnetic resonance (^1^H‐NMR) and carbon nuclear magnetic resonance (^13^C‐NMR). Whereas, the stereospecific action of enantiomer compound against the integrity of the cell membrane was visualized by confocal laser scanning microscopy with green fluorescence nucleic acid dye (SYTO‐9), and propidium iodide (PI). Moreover, bacterial cell morphology was observed through field emission scanning electron microscope. Lastly, the current study will also explore the effect of molecular chirality on the antioxidant capacity to further elucidate the enantioselective functional aspect of the enantiopure compound.

## 2 MATERIAL AND METHODS

### 2.1 Sample preparation

Plant material (ripe leaves of vine tea, 4‐Grade based upon the part of the plant classification) *Ampelopsis grossedentata* was collected from Moyeam Group Co., Ltd, from the region of Changsha, Hunan, China. The voucher specimen of *A. grossedentata* was confirmed and deposited at the College of Food Science and Engineering, Nanjing Agricultural University, Nanjing, Jiangsu, 210095, China. The collected material was air‐dried in darkness at room temperature and was stored in a air tight‐seal jar until needed.

Dried ripe leaves of vine tea (2017 production) plants (as mentioned above) were ground to make a fine powder. Fifty gram (50 g) dried, fresh, and sound leaves powder of vine tea with 8.53% moisture content on a dry matter basis (DB) was further used in the experiment. Racemate and enantiopure DHM standards were bought from Aladdin (Shanghai, China). HPLC grade ethanol and methanol were of HPLC‐grade used whereas, all other chemicals were of analytical grade. All the chemicals were correctly stored during handling, as mentioned for safety and storage instruction in the product manufacturer's instruction manual.

#### 2.1.1 Preparation of DHM by solvent extraction

First of all, fifty grams (50 ± 0.8 g dry matter based) vine tea powder was extracted with 1.5 L of metal‐free ethanol. After this, ultrasound mixing was done at 60°C for 25 min, followed by the filtration process. Then discard the sediments in collecting filtrates. The filtrates were further concentrated using a rotavap and then freeze‐dried using lyophilizer to get 7.441 g of compound A.

#### 2.1.2 Preparation of DHM by the crystallization process

**FIGURE 1:**
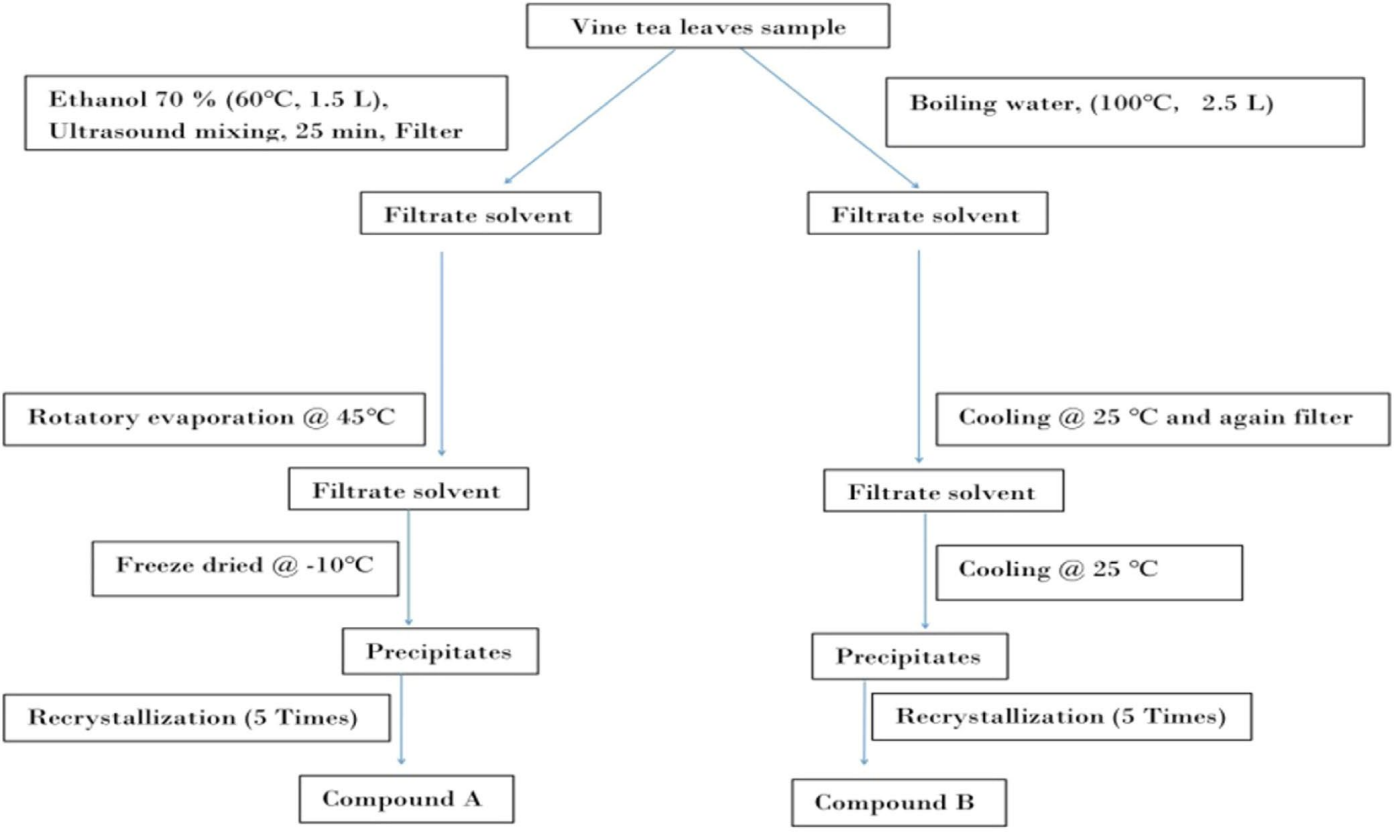
Isolation of Compound A (−)‐DHM and the (±) DHM (Compound B) from the leaves of *A. grossedentata*

Fifty grams (50 ± 0.8 g dry matter based) of dried vine tea leaf powder were extracted with 2.5 L distilled water for 8 hr and then filter to collect the filtrate. The filtrate was further settled down for two more hours and repeated the filtration process. This final filtrate was further precipitated by hot deionized water until the recrystallization process forms white crystals (repeat the recrystallization process five times) and then collected the crystals and weighted them before drying (33.24 ± 0.12 g). After drying, about 2.66 g of dry matter of compound B was obtained. The schematic diagram for the extraction of (−)‐DHM enantiopure, and the racemate (±) DHM by solvent extraction and recrystallization process, respectively, as depicted in Figure 1. After this, the identification of these two isolated compounds A and B from vine tea extract was carried out.

### 2.2 HPLC‐ESI/MS analysis

The extract was analyzed for further separation using high‐performance liquid chromatography (Agilent Technologies, Santa Clara, USA) with diode array detector (DAD) coupled with electrospray ionization mass spectrometry (HPLC‐ESI/MS). For HPLC‐DAD analysis, the sample was filtered through a filter‐membrane (0.45 µm, 13 mm). Ten microlitre volume of sample was injected into separating column Agilent Poroshell HC‐C18 (OBDTM, 250 mm × 4.6, 5 µm), with a mixture of solvent A; 0.1% acetic acid and solvent D; acetonitrile with 9:1 as a mobile phase. The gradient program was conducted for 55 min by following conditions: 0–10 min, 0%–40% A; 10–15 min, 40%–50% A; 15–25 min, 50%–75% A; 25–30 min, 75%–90% A; 30–40 min, 90%–100% A; 40–45 min, 100%–30% A; 45–50 min, 30%–2% A; 50–55 min, 2% isocratic; whereas, the other conditions of an experiment are as followed: the flow rate of mobile phase was adjusted to 0.8 ml/min, the elution was monitored at 289, 365 nm, with peak width 0.2 min and 1.25 scan/s, as described (Park, Kim, Rehman, Na, & Yoo, 2016). The conditions of mass spectrometry were as followed: the column and conditions were same as mentioned in HPLC‐DAD expect for injection sample volume (20 µl), the spray voltage source was set 3.0 kV and a heated transfer capillary temperature was set to 350°C and temperature of argon vaporizer was 200°C. The sheath gas was 40 arbitrary units, and auxiliary gas was set to 5 arbitrary units with a collision pressure of 1.0. The peak identification was performed in a positive and negative mode, and mass spectra (m/z) were recorded in the range of 100–1200. For LC‐ESI‐QTOF/MS, A TSQ‐Quantum Access MAX LC/MS (Thermo, USA) system was used. High‐resolution mass spectrometry analysis was carried out with Agilent 1,200 HPLC system and 6,410 QTF‐MS system (Agilent Technologies, Santa Clara, USA) with heated electrospray ionization (ESI) source.

### 2.3 NMR analysis

Nuclear magnetic resonance (NMR) (Bruker, Fallanden, Switzerland) ^1^H and ^13^C NMR spectral data of DHM in CH_3_DO, detected at 500.130 MHz and 125.758 MHz, by gradient equipped inverse 5 mm triple probe with Pi/2 pulses of 6.3 and 14.2 microseconds, respectively.

### 2.4 Quantitative analysis

For the calibration curve, commercially available DHM (as mentioned in the sample preparation section) was used to prepare a stock solution of various concentrations (0.0012–1.002 mg/ml).

### 2.5 Antioxidant capacity

#### 2.5.1 DPPH radical‐scavenging ability

The DPPH radical‐scavenging ability of racemate DHM was measured in comparison with enantiopure DHM, such as (+) DHM and (−) DHM (Li, Ji, et al., 2014) with slight modification. The determination of the antioxidant ability of the compound depends on the ability of a compound for donating a hydrogen atom to eliminate DPPH radical, as reported (Suttirak & Manurakchinakorn, 2014). The stock solution of ascorbic acid with various concentrations was prepared after dissolving in deionized water and used as a reference sample. Of 0.5 ml DPPH solution (0.4 mM, ethanol), 1.0 ml sample (DHM) was added in the glass test tube and added 2.0 ml water. After mixing the test tube vigorously, incubated it for 30 min and measured the absorbance at 517 nm using a UV‐spectrophotometer. Then, the scavenging activity % was calculated by following Equation 1.

(1) $$\text{Scavenging power}(\%)=\left(1-\frac{\text{Asample}-\text{A0}}{\text{Ablank}}\right)100$$

A_0_ can be determined by following the above procedure, but the only change was deionized water instead of the DPPH solution. The blank reading was noted as A_blank_, measured by observing the absorbance values at 517 nm (with the addition of the sample).

#### 2.5.2 Reducing power ability

The ability of compound for reducing power was determined by the method (Wang et al., 2014) with slight modification, and ascorbic acid was used as a positive control. Simply, 2.5 ml sample was mixed with 2.5 ml of 0.1% phosphate ferricyanide (w/v) and 2.5 ml of 0.2 mM phosphate buffer (pH = 6.62) in the glass tube and heated the glass tube mixture at 50°C for next 20 min. Then, 2.5 ml of 10 % of trichloroacetic acid (w/v) was added. Then, the above‐prepared mixture was centrifuged for 10 min at 6,000 × g. After this centrifugation process, supernatants were collected and discarded the sediments. Then 5 ml of supernatant was added into a separate tube and mixed with 0.5 ml of 0.1% ferric chloride (w/v). Absorbance was determined at 700 nm using a UV‐spectrophotometer (Shimadzu Co., Kyoto, Japan).

### 2.6 Antimicrobial activity

Agar well diffusion method was used with slight modification for antimicrobial activity of (+) DHM, (−)‐DHM, and (±) DHM (Valgas, Simone, Elza, & Smania, 2007). The assay was carried out in triplicates, and then, measurements of inhibition zone (ZOI) were taken from the top of the well to the clear zone in millimeters (mm).

#### 2.6.1 Determination of MIC and MBC

The minimum inhibitory concentration (MIC) and minimum bactericidal concentration (MBC) were determined by using two serial dilution methods (Zhao, Hong, Dong, Meng, & Mu, 2013) with minor modification. The antimicrobial agent was mixed with Tween 80 (5% w/v). Twofold serial dilution of Tween 80 prepared in sterile nutrient broth (NB) medium ranging from 16 μg/ml to 256 µg/ml. The same concentration and volume of bacterial strains were inoculated with (+) DHM, (−) DHM, and (±) DHM in 96‐well microplates. Then, visual turbidity was noticed within the plates after incubation at 37°C for 24 hr. However, no growth was observed after subcultured 10 ml of the MIC solution on potato dextrose agar (PDA) at 37°C for 24 hr in minimum bactericidal concentration (MBC).

#### 2.6.2 Determination of Cell membrane integrity of *B. cereus* (AS11846)

The integrity of the bacterial cells was observed by determining the leakages of the constituents in the supernatants, as described by (Cui, Zhang, Zhou, Zhao, & Lin, 2015). First of all, the supernatant was collected for each sample, and then, the absorption was noticed at 260 nm (OD_260_) at a serial of time intervals. The exponential phase (1 × 10^7^ log CFU/ml) bacterial cells of *B. cereus* (AS11846) harvested and centrifuge at 5,000 × g for 5 min at 4°C and put in phosphate buffer (PBS 0.1 M, pH 7.0). Consequently, the microbial cells were treated with (32, 64 μg/ml) for 1, 2, 4, and 8 hr, whereas the bacterial suspension in the absence of DHM was treated as a control.

#### 2.6.3 Confocal laser scanning microscopy (CLSM)

The viability of *B. cereus* (AS11846) bacterial cells were also observed through confocal laser microscopy after the treated/untreated with DHM enantiopure. The bacterial suspension (1 × 10^7^ log CFU/ml) was treated with 32 μg/ml of (±) DHM, and (−)‐ DHM and then incubated at 37°C for 24 hr. The treated/untreated bacterial cells were further stained with propidium iodide (PI) and Green Fluorescence Nucleic Acid (SYTO‐9) dyes to observed cell viability through confocal laser microscopy (TCS‐SP, Leica, Germany) as described by the method (Sun, Dong, Sun, Ma, & Shang, 2015).

#### 2.6.4 Determination of physical damage on the cell membrane

Scanning electron microscopy analysis (*SEM*) was performed according to the method of (Li, Xing, et al., 2014). The *B. cereus* (AS11846) cells suspensions were incubated with 32 μg/ml (1 × MIC), 64 μg/ml (2 × MIC) of (+) DHM, (−)‐DHM, and (+)‐DHM for 1, 2, 4, and 8 hr and then centrifuged (Eppendorf centrifuge 5804R AG, Hamburg, Germany) 5,000 × g at 4°C for 5 min and resuspended in phosphate‐buffered saline (PBS; pH, 7.0). Then washed the pallets three times with buffer and reacted with isolated compounds until reaching the final concentration up to 32 µg/ml, and 64 µg/ml and then transfer onto the coverslip. The prepared sample without the addition of DHM was treated as a control. Slide‐immobilized cells were fixed with glutaraldehydes (2.5% w/v) solution. The physical damage to the cell membrane was noticed through SEM (SU‐8010, Hitachi, Tokyo; Japan) at 30 kV of the electric beam.

### 2.7 Statistical analysis

The data were statistically analyzed as a mean of standard deviation (±*SD*). One‐way analysis of variance (ANOVA) and the Student *t* test was used to analyze the data at *p* < .05 significance level. All the data were analyzed in triplicate by Statistical Packages for Social Sciences (SPSS) 17.0.5 version.

## 3 RESULTS AND DISCUSSION

### 3.1 Dry matter contents

Five grams of the vine tea powder was weighed and hot air‐dried at 103 ± 2°C for at least 16 hr in an oven to get constant weight and repeated the experiment three times.

### 3.2 Semi‐preparative separation of DHM enantiomer

The development of a high‐efficient extraction method should consider both the quality and quantity of isolates. For the further identification of the physical properties of the compound solution stability test analysis was carried out. Results indicated that (−)‐DHM was found to be more soluble in ethanol than water, and it reaches 165–170 mg/ml at room temperature. Moreover, in the case of water as a solvent to dissolve (−)‐DHM, the finding showed that the sample (−)‐DHM was more soluble in hot water (15–18 mg/ml at 75–80°C) than cold water (2–3 mg/ml at 25°C). The Better absorption of (−)‐DHM was showed in ethanol than water because the ethanol‐dihydromyricetin adduct possesses higher acidity than water‐dihydromyricetin, as reported earlier (Kalíková, Martínková, Schmid, & Tesařová, 2018; Lesellier & West, 2015). So results proved that (−)‐DHM has better absorption in the organic solvent than water. It is noteworthy to mention that (−)‐DHM can be easily oxidized by high‐temperature exposure or/and direct in‐contact with the metal surface. As a result, the racemization process may increase the quantity of the racemate mixture (±) DHM and affect the quality of desire enantiopure (−)‐DHM. A similar finding has been reported by another researcher, where the author has found the acceleration of the racemization process at a higher temperature and metal concentration (Qingquan, Yuan, & Zeng, 2014; Wang, Xiong, & Fang, 2016).

#### 3.2.1 HPLC‐ESI/MS Analysis for isolates

**FIGURE 2:**
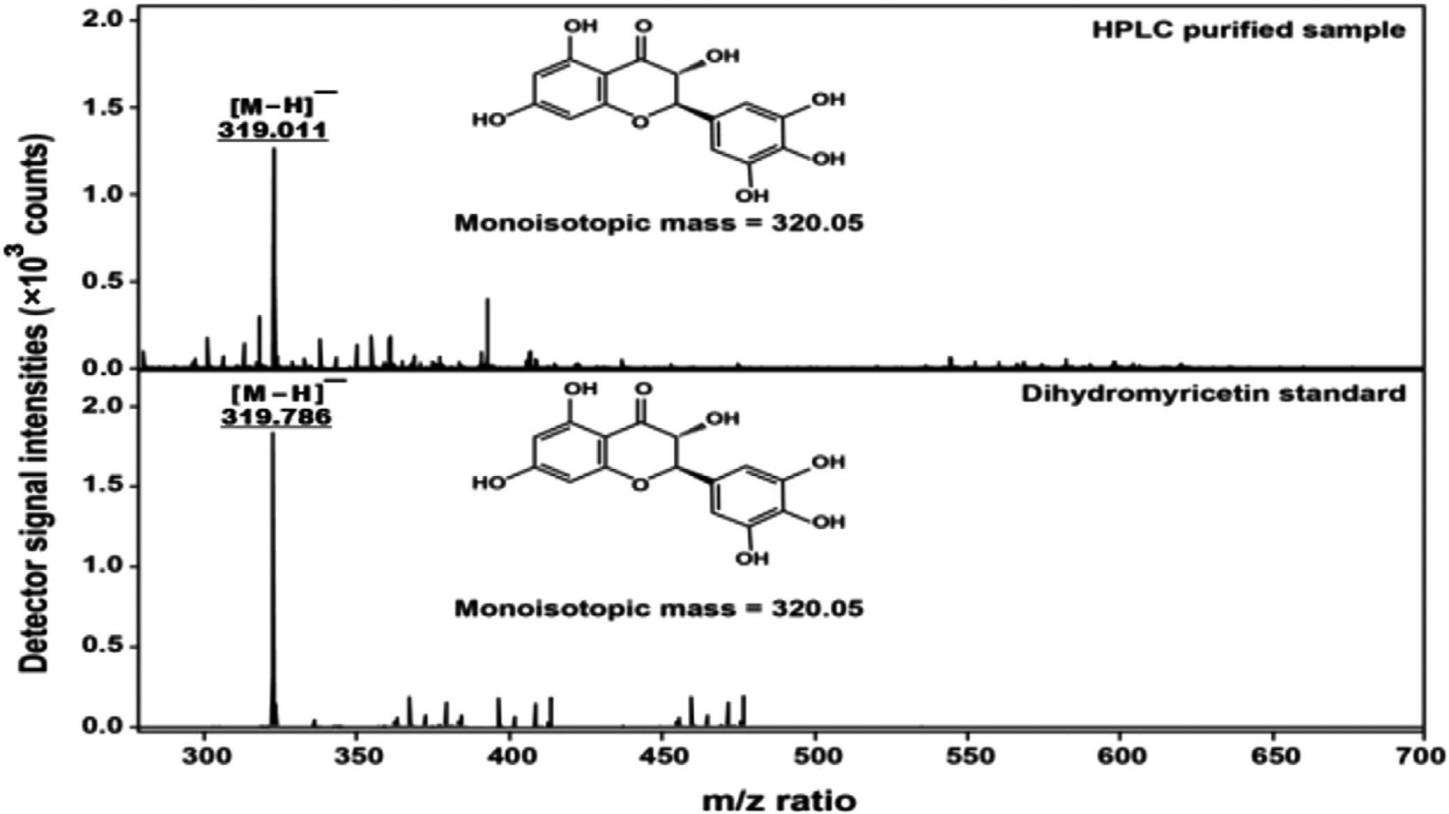
The obtained HR‐ESI/MS of (−)‐DHM, using an HPLC‐purified sample after extraction (top) in comparison with (−)‐DHM commercially available standard (bottom)

The isolation of compounds A and B was achieved successfully at 365 nm. The structure of compound A was identified as enantiopure dihydromyricetin (−)‐DHM, whereas compound B was a racemate mixture of (±) DHM. The findings of the current study were in line with the previously reported work (Kalíková et al., 2018; Lesellier & West, 2015). The content of compound A and B were determined and quantified as 32.96 ± 1.18%, 77.15 1.98% (*n* = 3) from HPLC‐ESI/MS analysis, and by comparing with commercially available their respective standard. Figure 2 explains the results obtained from HR‐ESI/MS spectra of (−)‐DHM using an HPLC‐purified sample after extraction (top) in comparison with (−)‐DHM commercially available standard (bottom).

**FIGURE 3:**
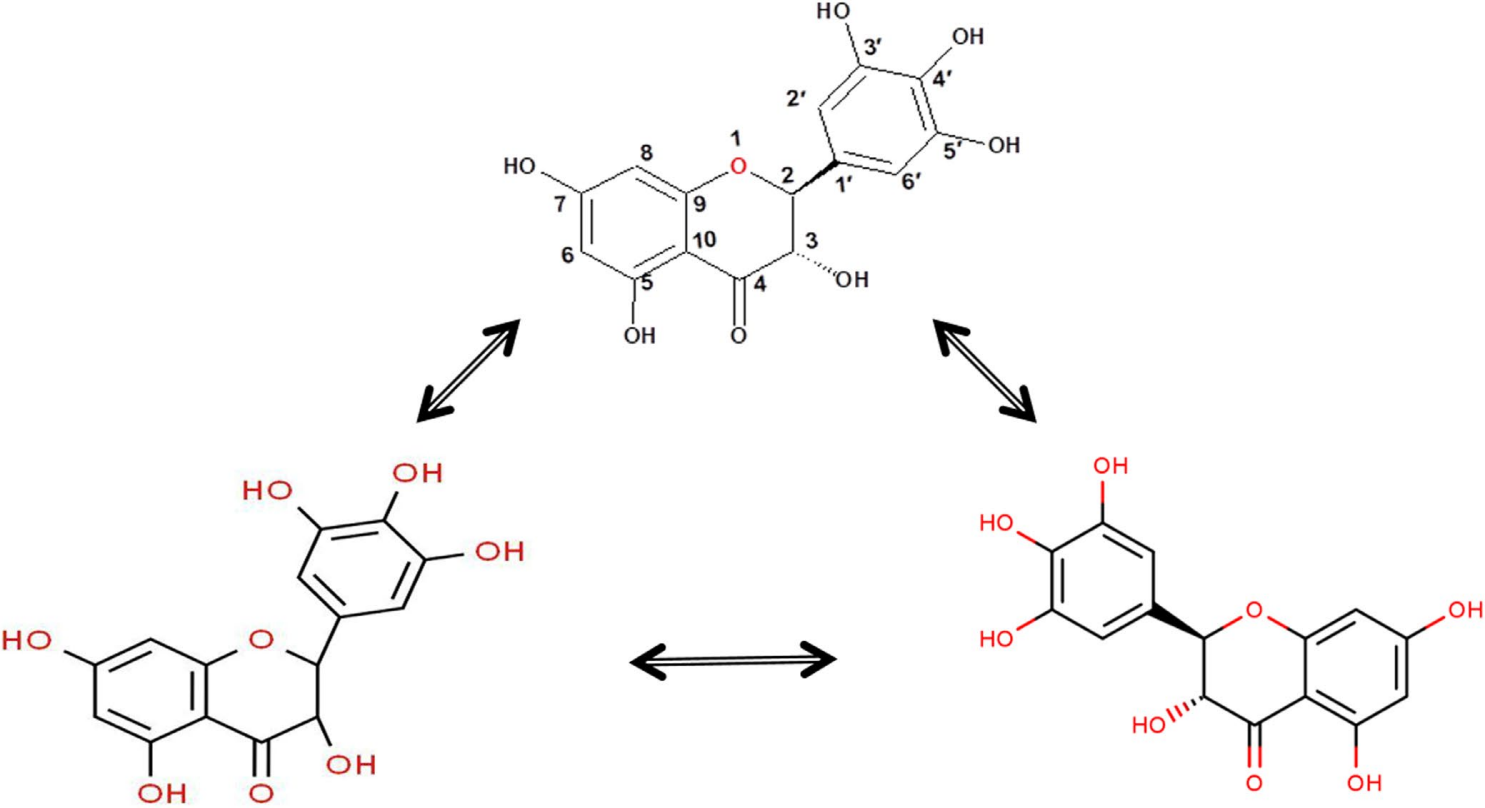
Molecular structure of dihydromyricetin (DHM) and its possible enantiopure compounds

In comparison to our study, Du and Gao also have reported the (−)‐DHM extraction method at a high purity of over 99% with a preparatory of triple‐column counter‐current chromatography. However, this method is challenging to put in industrial use (Du, Cai, Xia, & Ito, 2002; Zhu et al., 2019). However, the extraction method optimized by HPLC analysis of (−)‐DHM has 95% purity in this case, and it could be obtained by the re‐crystallizing process of vine tea leaves for five times (as depicted in the method section Figure 1), which was a useful technology for industrialization. The extraction process adopts in the current study is much more proficient and controllable than that of the prevailing process. Therefore, the finding of the current study can be used as a base for analytical scale to semi‐preparative scale study, which further can be explored for the industrial scale. Du and Gao have reported a similar finding when the chiral separation of DHM has been done by using supercritical fluid chromatography and keeping the view of the influence of different chiral separating solvent, co‐solvents, and the flow rate (Speybrouck & Lipka, 2016; Wang et al., 2016). Moreover, the presence of two chiral‐centers (C‐2 and C‐3) in the molecular structure of dihydromyricetin (DHM) and its possible two enantiopure compounds has been explained in Figure 3.

#### 3.2.2 Identification and purity analysis of enantiomer of DHM by NMR spectroscopy

**FIGURE 4:**
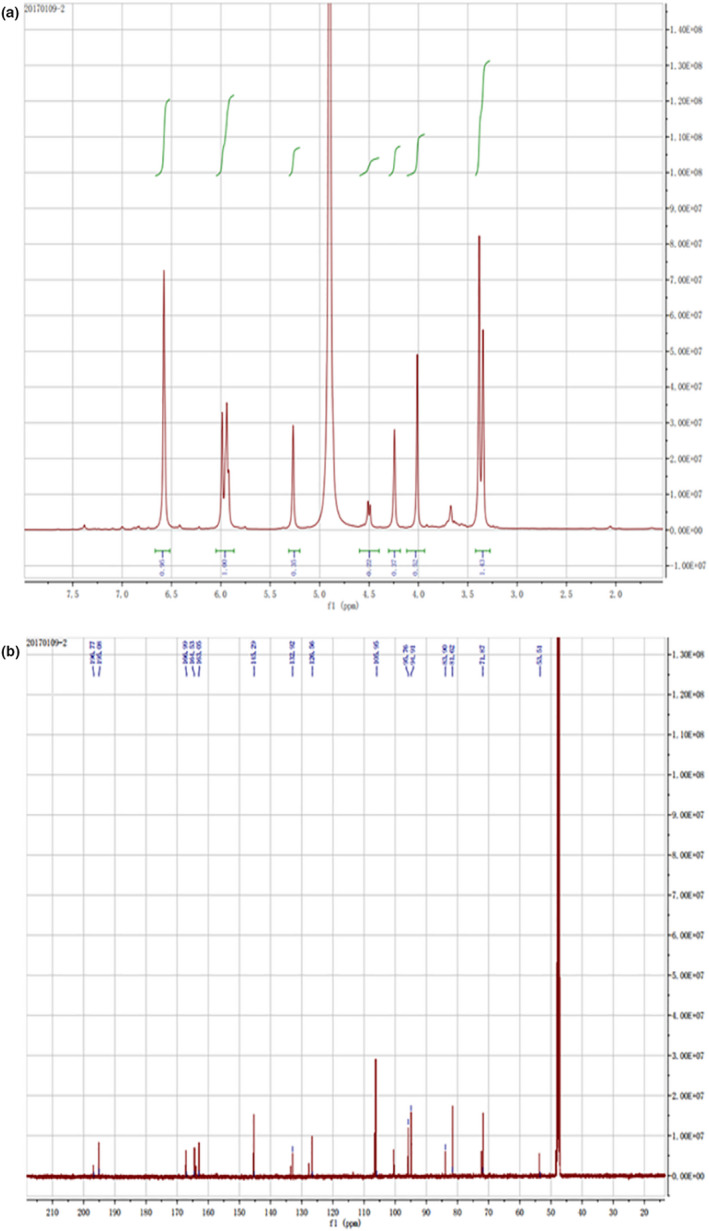
(a) NMR spectrum for 1H and (b) NMR spectrum for 13C

The identity and purity of homemade and commercially available enantiopure DHM were further tested through UV, ESI‐MS, and NMR analysis. A typical chiral NMR spectrum for chiral (−)‐DHM is shown in Figure 4. The identification of the isolated compound B was characterized as 639.0 [2M−H]^−^ (13.5); 355 [M+Cl]^+^ (18.9); 319.1 [M−H]^−^ (1.0), while compound A as a (−)‐DHM was identified by ^1^H NMR analysis. In our laboratory, the ^1^H NMR recorded at ^1^H NMR (400 MHz, CDCl_3_), we observed the improvement of the compound [(−)‐DHM)] more than 40% (26 mg/100 g). The ^1^H NMR analysis disclosed that the proton of the hydroxy‐methylene group (5C‐OH) nearby the carbonyl group of 2 has the chemical shift around 11.87 ppm (C7‐OH) at 10. 81 ppm, while the C3′‐OH which appear with a chemical shift around 8.90 ppm, C5′‐OH has singlet peak at 8.20 and C4′ at 6.39 ppm, while 3 have the chemical shifts of the 6 (CHOH) bond between 5.3 and 5.5 ppm (Figure 4a). The ^1^H NMR spectrum also shows the single doublet peaks in a phenyl ring; the C6‐H at 6.39 ppm, the doublet peak of C8‐H at 5. 89, while the chemical shift of the protons on the C2′ and C6′ appear with doublet peaks at 4.90 and 4.41 ppm, respectively. Further detailed result of (−)‐DHM, ^13^C NMR are shown in (Figure 4b). ^13^C NMR: 196.77 (C‐4), 166.997 (C‐7), 163.05 (C‐5), 161.531 (C‐9), 145.299 (C‐3′and C‐5′), 132.925 (C‐4′), 126.553 (C‐1′), 106.950 (C‐2′ and C‐6′), 100.902 (C‐10), 95.760 (C‐6), 94.910 (C‐8), 83.903 (C‐2), and 81.620 (C‐3). The finding of this study is in line with the finding of other researchers, where the authors have studied a similar common chiral (Chaturvedula, Chen, Yu, & Mao, 2013; Chaturvedula & Huang, 2013). We are herewith reporting the successful isolation of (−)‐DHM enantiomer based on extensive (^1^H and ^13^C). These findings confirmed that the isolated compound is enantiopure DHM with the desired enantiomeric excess level.

### 3.3 Antioxidant properties of enantiopure DHM

#### 3.3.1 DPPH radical‐scavenging ability

**FIGURE 5:**
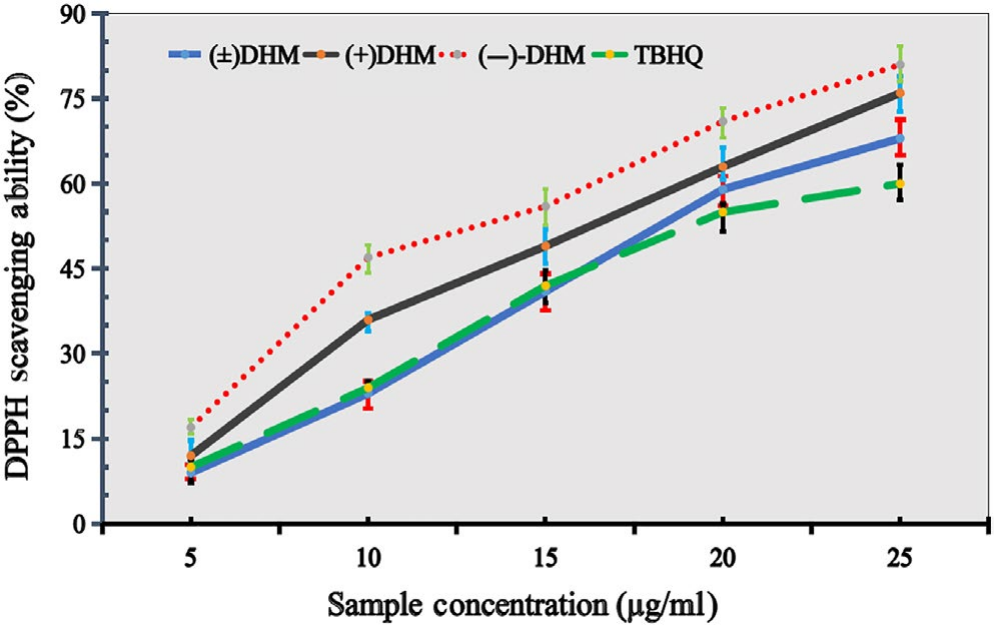
DPPH scavenging ability (%) of (+) DHM, (−)‐DHM, and (±) DHM in comparison with synthetic antioxidant (TBHQ)

The DPPH radical‐scavenging ability was measured at 517 nm, as mentioned in the method sections. A higher DPPH radical‐scavenging activity was observed for both of (−)‐DHM and (±) DHM samples in a concentration manner. The finding of the present study depicted that (−)‐DHM could reduce oxidative damage. A similar observation of dihydromyricetin for preventative oxidative damage has been reported in previous studies, where the author has proposed that DHM could follow one of the following mechanism to reduce oxidative damage mechanism such as by direct radical‐scavenging mode, acting as Fe^2^‐chelation and increasing the superoxide dismutase (SOD) enzyme activity which mainly catalyzes the dismutation of superoxide anion O_2_(−) to molecular oxygen (Song et al., 2017), and/or by activating phosphatidylinositol 3‐kinase (PI3k/Akt) and modulating the nuclear transcriptional factor‐erythroid 2‐related factor 2 (Nrf2), that take part in antioxidant activity and gene encoding detoxification (Hu, Zhang, Yi, Zhou, & Mi, 2018; Luo et al., 2017). More interestingly, the finding of our study also suggested that enantiopure (−)‐ DHM (compound A) have better antioxidant ability than that of (+)‐DHM (compound B). It might be due to the better efficacious behavior of enantiopure compounds over racemates mixture. Thus, enantiopure (−)‐ DHM (2S, 3S)‐3, 5, 7, 3′, 4′, 5′‐hexahydroxyflavanone) have better functional property than racemic mixture (±) DHM, as shown in Figure 5. Also, this better antioxidant capacity of enantiopure compounds over racemates might be due to the enantioselective properties of enantiopure compounds that normally vary from racemate mixture. Further, it is also documented that the chirality of the biological macromolecules results in stereospecific action and different affinities of enantiomers (Hu et al., 2018), which may result in the improvement of its functional properties. However, it is further suggested that research should be done for detail studied to understand enantiopure superior or inferior mechanism, along with its stability and other functional aspects. DPPH scavenging ability (%) of (+) DHM, (−)‐DHM, and (±) DHM in comparison with synthetic antioxidant tert‐Butylhydroquinone (TBHQ) were seen in following order of activity (−)‐DHM> (+) DHM> (+)‐DHM > TBHQ.

#### 3.3.2 Reducing power capacity of DHM enantiomer

**FIGURE 6:**
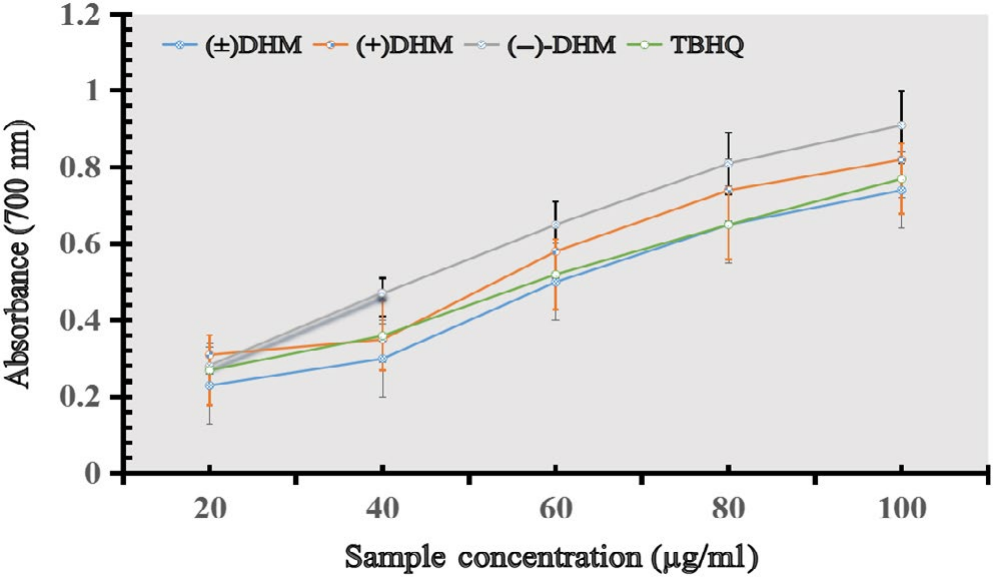
Reducing power capacity of (+) DHM, (−)‐DHM, and (±) DHM in comparison with synthetic antioxidant (TBHQ)

The reducing power can serve as a significant parameter to reflect the antioxidant activity of a compound. The reducing power was determined at 700 nm by the method (Zou, Lu, & Wei, 2004). All samples showed different degrees of reducing power. Noticeably, the reducing power of (+)‐DHM and (−)‐DHM was comparable to the commercially available synthetic antioxidant TBHQ used as the food industry (Figure 6). The findings of the current study are similar to the conclusion of the previously reported studies (Gao et al., 2009).

### 3.4 Antimicrobial activity of enantiopure DHM

The different concentration of enantiopure (−)‐DHM has demonstrated the efficient inhibition activity against selected foodborne pathogen such as *B. pumilus* (CMCC63202), *B. cereus* (AS11846); *E. coli* (ATCC25922); *S. aureus* (ATCC25923), and *P. fluorescens* (AS11802) particularly against *B*. *cereus* (AS11846) with 25.42 ± 0.26 mm inhibition zone (ZOI). The different concentrations of the (+)‐DHM, (+) DHM, and (−) DHM were further tested to determine minimum inhibition concentration (MIC), minimum bactericidal concentration (MBC), and the zone of inhibition (ZOI) against abovementioned foodborne pathogens Table 1. Results indicated that the MIC values for *S. aureus* (round‐shaped, Gram‐positive) and *E. coli* (rod‐shaped, Gram‐negative) were found as 64 μg/ml and 32 μg/ml, respectively. Nisin (polycyclic antimicrobial peptide commonly used as a preservative in the food industry) was used as a positive control. Also, the antibacterial activity of (−)‐DHM was time‐ and dose‐dependent against pathogen strains. The effectual antimicrobial activity of (−)‐DHM enantiopure against Gram‐positive and Gram‐negative might be due to the presence of hydroxyl groups (OH^−^) on the 5th and 7th position around the carbon structure, because it has been demonstrated that hydroxyl group at 5th and 7th positions are implicated in the antimicrobial character. A similar finding has been observed in the previously, where the author had proposed the hydroxylation at 5th and 7th carbon, in the flavonols, are critically involved in the antimicrobial property (Echeverria, Opazo, Mendoza, Urzua, & Wilkens, 2017). Therefore, the presence of a hydroxyl group at carbon 5th and 7th carbon position could be a reason that makes (−)‐DHM unique over the other flavonoids. A similar structural interaction of flavonoids and its antimicrobial activities was determined in previous reports, where the author had investigated and proposed a similar relationship of hydroxylation at carbon 5th and 7th positions with their antimicrobial properties (Liu, Du, Zeng, Chen, & Niu, 2009). This is further supported by another study, where the finding of the study revealed that the steric‐effect of a compound could enable it for improved antimicrobial activity (Hongxia, Jing, Jiayi, Wenjing, & Jianhao, 2019). That is why the present study has proposed the unique antimicrobial mechanism of (−)‐DHM enantiopure as compared to racemate (+)‐DHM involved against foodborne pathogens.

**TABLE 1:** Antimicrobial activity of (+)‐DHM, (+)DHM, and (−)‐DHM isolated from vine tea

Bacteria	Control (Nisin)			(±)DHM			(+)DHM			(−)‐DHM		
	ZOI	MIC	MBC	ZOI	MIC	MBC	ZOI	MIC	MBC	ZOI	MIC	MBC
	(mm)	(µg/ml)		(mm)	(µg/ml)		(mm)	(µg/ml)		(mm)	(µg/ml)	
*B. pumilus* CMCC63202	13.47 ± 0.41^c^	64	64	16.18 ± 0.20^e^	64	64	20.68 ± 0.16^d^	64	64	21.37 ± 0.12^d^	32	64
*B. cereus* AS11846	14.12 ± 0.67^b^	64	64	21.51 ± 0.25^a^	32	64	23.34 ± 0.41^a^	32	32	25.42 ± 0.26^a^	32	32
*E. coli* ATCC25922	NA	NA	NA	19.88 ± 0.22^c^	64	64	22.23 ± 0.43^b^	32	64	24.21 ± 0.10^b^	32	32
*S. aureus* ATCC25923	15.5 ± 0.26^a^	64	64	17.12 ± 0.41^d^	64	64	20.22 ± 0.12^d^	32	64	21.50 ± 0.17^d^	64	64
*P. fluorescens* AS11802	NA	NA	NA	20.33 ± 0.32^b^	32	64	21.66 ± 0.13^c^	32	64	22.12 ± 0.25^c^	32	64

Note

All the values represent the mean of three independent experiments ±*SD* whereas, values with different letters (a‐e) in the same column are significantly different (*SD*) from each other.

#### 3.4.1 Integrity of the cell membrane

The integrity of the *B*. *cereus* (AS1.1846) cell membrane was further assessed after treated/untreated with different concentration (32 µg/ml (1 × MIC) and 64 µg/ml (2 × MIC)) of (−)‐DHM. The absorbance of the supernatant was recorded at 260 nm. Results indicated that a significant increase in the absorbance values (OD_260_) of supernatants was noticed after treating with (−)‐DHM at the concentration of 1 × MIC. The reason for increasing the OD_260_ values after treating with (−)‐DHM is due to the capability of (−)‐DHM to increase the cell membrane permeability, which can damage the cell membranes. Consequently, leakage of internal cell constituents may occur into the supernatants and may increase the absorbance (OD_260_).

Furthermore, it is also well documented that any increases in the absorbance (OD_260_) values reflected the leakage of essential material (nucleic acid and proteins) from the cells and showed the alteration of membrane permeability (Peng et al., 2020). A similar dose‐ and time‐dependent antimicrobial activity of dihydromyricetin has been reported earlier, where the author has reported the increase in OD_260_ values and suggested that this is because of the leakage of internal constituents from the cell through the affected membrane (Xie, Yang, Tang, Chen, & Ren, 2015). However, a noticeable increase was showed in OD_260_ values of the supernatant in the case of 2 × MIC, which shows a quick response and a big jump in OD_260_ when treated for 8 hr at 2 × MIC (Table 2). That is why incubation for 8 hr caused a severe lethal effect on the cell membrane, which eventually causes the complete disruption of the cell membrane and release of all internal cellular material into the supernatant and causes cell death. However, in the case of the untreated cells, no significant difference was seen in the OD_260_ value even incubated for 8 hr. So, these results indicated (−)‐DHM that could increase the permeability of the cell membrane and cause the leakage of intercellular contents (nucleic acid and protein) through the damaged cell membrane and accounting for the increased in the concentration at 260 nm. This dose‐dependent antimicrobial activity was also observed and reported by a researcher previously (Ameen, Alyahya, Bakhrebah, Nassar, & Aljuraifani, 2018). Therefore, it suggested that (−)‐DHM can disrupt the cell membrane and causes the leakage of the essential material from the cell and finally causes the cells death. These results further tested and confirmed through the observation of the morphological changes after reacting with (−)‐DHM through *SEM* and CLSM.

**TABLE 2:** Cell membrane leakage measuring at OD260 after treating with (−)‐DHM against *B. cereus* (AS1.1846) (*n* = 3)

_Cell constituent's release OD260 values_	_(+)‐DHM_				_(+)DHM_				_(−)‐DHM_			
	_1 h_	_2 h_	_4 h_	_8 h_	_1 h_	_2 h_	_4 h_	_8 h_	_1 h_	_2 h_	_4 h_	_8 h_
_Control_	_0.09 ± 0.02_	_0.10 ± 0.02_	_0.13 ± 0.04_	_0.14 ± 0.01_	_0.10 ± 0.04_	_0.12 ± 0.02_	_0.13 ± 0.08_	_0.14 ± 0.04_	_0.08 ± 0.06_	_0.12 ± 0.01_	_0.11 ± 0.02_	_0.13 ± 0.01_
_1 × MIC_	_0.11 ± 0.02_	_0.14 ± 0.03_	_0.19 ± 0.01_	_0.23 ± 0.02_	_0.15 ± 0.03_	_0.18 ± 0.04_	_0.25 ± 0.05_	_0.28 ± 0.02_	_0.19 ± 0.02_	_0.21 ± 0.05_	_0.28 ± 0.06_	_0.31 ± 0.05_
_2 × MIC_	_0.18 ± 0.03_	_0.21 ± 0.02_	_0.25 ± 0.04_	_0.29 ± 0.01_	_0.21 ± 0.01_	_0.23 ± 0.01_	_0.31 ± 0.05_	_0.34 ± 0.06_	_0.26 ± 0.05_	_0.29 ± 0.03_	_0.37 ± 0.03_	_0.39 ± 0.04_

#### 3.4.2 Determination of morphological changes in *B. cereus* through *SEM*

**FIGURE 7:**
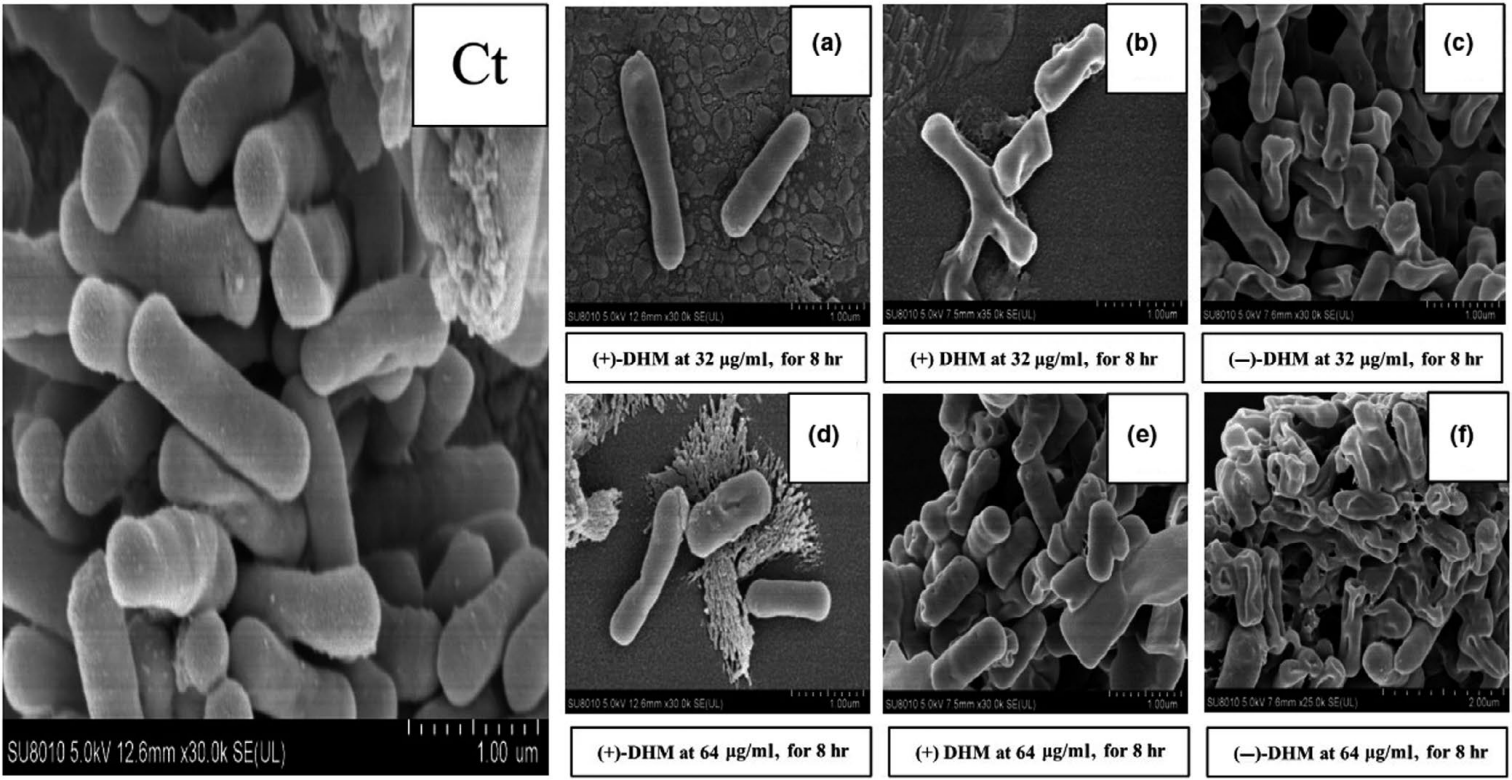
The effects of (−)‐DHM on *B. cereus* (AS11846) was observed with the help of a scanning electron microscope (*SEM*). 7‐*C*t represents the results of *B. cereus* (AS11846) strains when treated without a sample (Control), (a) represent the results of *B. cereus* (AS11846) strains when treated with (+)‐DHM for 8 hr at 32 μg/ml, (b) showed the data of (+)DHM for 8 hr at 32 μg/ml, (c) (−)‐DHMfor 8 hr at 32 μg/ml, d (+)‐DHM at 64 μg/ml for 8 hr, e (+) DHM at 64 μg/ml for 8 hr, and f (−)‐DHM at 64 μg/ml for 8 hr

Scanning electron microscopy (*SEM*) was further employed to explore the mode of action of (−)‐DHM against *B. cereus* (AS11846). The *SEM* observation showed the alteration in the morphology of the *B. cereus* (AS11846) cells. The morphological changes (cell membrane damage) in the *B. cereus* (AS11846) cell after treating/untreated with (+)‐DHM, (−)‐DHM, and (+) DHM are shown in Figure 7(a‐f). It is clear from the Figure 7‐*C*t that untreated *B. cereus* (AS11846) cells, nominated as a control, show the typical, rod‐shaped, smooth membrane, and striated cells structure (with intact outer surface), which further showed that the cells were active and alive in the control sample. In constant consideration for 8 hr, there was no significant difference in the shape and structure of the cell membrane. However, it might be possible that there is some difference in cell membrane permeability. As it is reported, the permeability of the bacterial cell membrane might have increased as time passes (Cui et al., 2015). In contrast, when the *B. cereus* (AS11846) bacterial cells were incubated with (+)‐DHM (32 μg/ml) for 8 hr, then irregularities, bleb projections, and concave formation were seen with more shrinkage that was started on the partial cell membrane (Figure 7a). A similar finding has been reported by another researcher (Anwar et al., 2019), where the impact of natural antimicrobial compounds (flavonoids) on the morphology of rod‐shaped bacteria has been observed. Moreover, when *B. cereus* (AS11846) bacterial cells were treated with (−)‐DHM at 1 × MIC for 8 hr then coarse surfaces, even membranes of the cells were further surrounded by blebs, irregularities, and even a portion of cells began to become flat and trans‐membrane ion holes were also formed which started to lose its original form (rod‐shaped) almost near to utterly flat with very less or almost no bleb anymore (Figure 7c). That is why the finding of the study suggested that the action mode involved might be due to changes in the cell permeability that can affect membrane protein and lipid interaction that disturb the ionic protein‐lipid bond, so ultimately, it affects the protein structure and ultimately its function. The finding of this study is in line with the previous study, where the author has explained that phospholipids are a significant component of the cell membrane and they have functional role particular acidic phospholipids because it is involved in the formation of micro‐domain of the plasma membrane and can ionically interact with protein via polybasic sequences (Li, Shi, Guo, Li, & Xu, 2014c).

#### 3.4.3 Determination of morphological changes in *B. cereus* (AS11846) through CLSM

**FIGURE 8:**
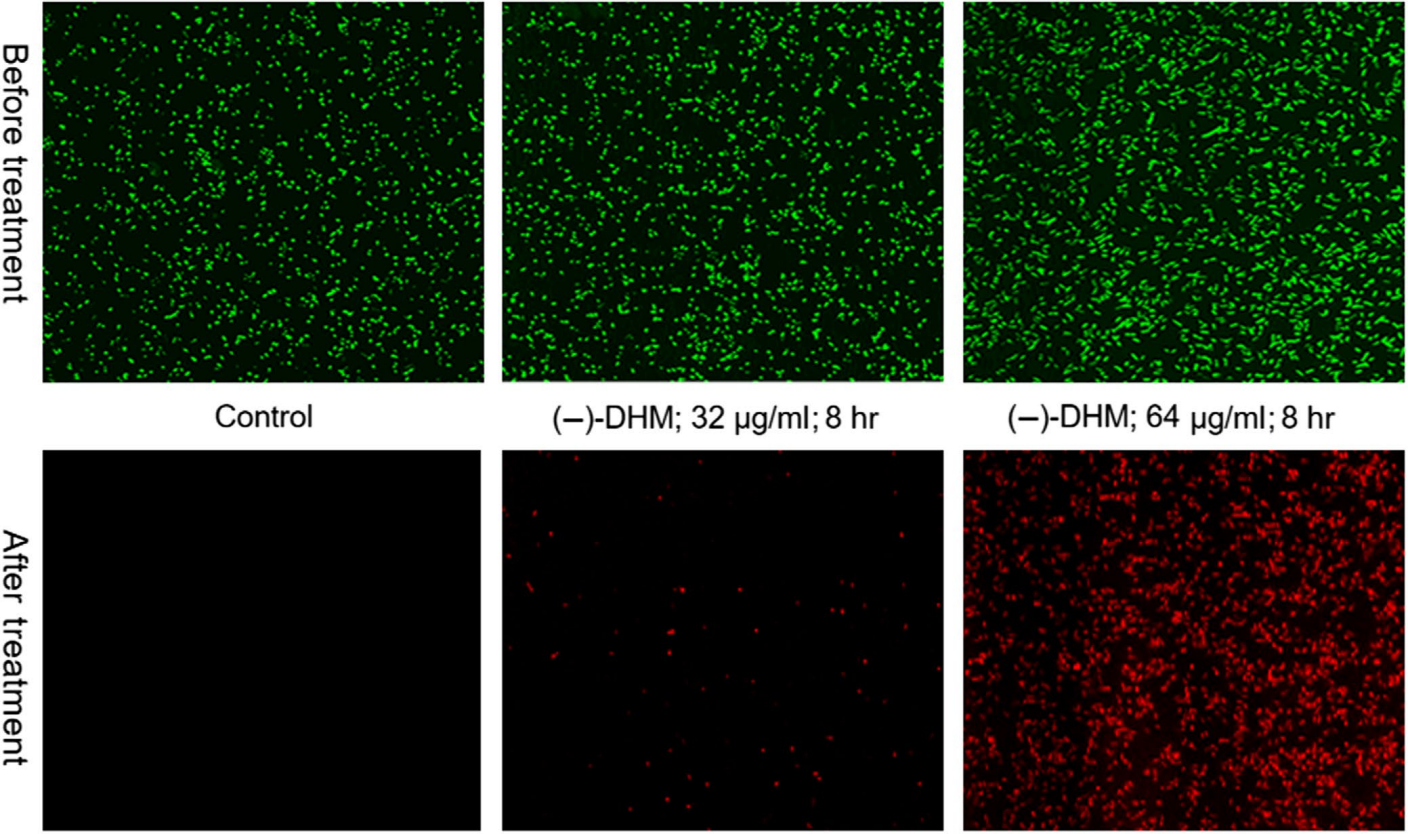
Confocal laser scanning microscopy image of *B. cereus* (AS11846) treated with control (left corner); (−)‐(DHM) at 1 × MIC (Middle one), and 2 × MIC for 8 hr (right corner)

Confocallaser scanning microscopy (CLSM) was further used to explore the action mode of enantiopure (−)‐DHM involved in antimicrobial activity. The finding of the current study suggested that the (−)‐DHM can increase cell membrane permeability and cause injury of the microbial cell surface, which releases essential internal constituents and thus causes cell death, as can be seen in Figure 8. Also, better penetration capacity of PI with more prolonged exposure of time (8 hr) produced red color after PI could enter into double‐stranded DNA through the damaged cell membranes. This red color indicated the rigorous injury by the action of (−)‐DHM on the cell membrane, while SYTO‐9 could enter through the cell membrane to bind the DNA of live cells and produce green fluorescence (Sun et al., 2015). It is noted that the SYTO‐9 stains will cover the intact cell membrane bacteria and produce green fluorescence, whereas; PI will penetrate through the decade cell membrane of dead bacteria and produce red fluorescence. Therefore, we suggest that this work has the potential for the development of chiral bacteriostatic material because stereo‐configuration will provide insight into the development of novel antimicrobial compounds with better biosafety.

## 4 CONCLUSION

The findings of the current study suggested that vine tea leaves are rich in dihydromyricetin. The compound (−)‐DHM, was found to be more potent antimicrobial and antioxidant as compared to synthetic antimicrobial (nisin) and antioxidant agents (TBHQ). Among the selected bacteria, (−)‐DHM exhibited preferred antibacterial activity against *B. cereus (AS11846)*. Cell structure analysis of *B. cereus* (AS11846) revealed that (−)‐DHM could increase the cell permeability and cell leakage, causing cell death at the end. These results also suggested that the enantiopure (−)‐DHM can be used to certify the bacteriological safety. Though, additional work is needed, prior to the application of the enantiopure, (−)‐DHM in food processing and preservation industries such as process optimization, the interaction of DHM with other food components and whether this enantiopure characteristic is capable of inducing microorganism into viable but nonculturable (VBNC) form along with the sublethal injury. Studies of the cell organism also need to be further explored. No doubt, the use of the most synthetic chiral drug was not accepted critically due to the high cost involved in the separation of stereoisomers on an industrial scale. However, recent progress in the present work will aid in the chemical technology to simplify the separation and preparation of enantiopure and the significance in this research area. In short, it is concluded that the (−)‐DHM has a strong potential to be used as an alternate of the synthetic antioxidant and antimicrobial agent in food and other phytopharmaceutical industries.

## 5 ETHICAL REVIEW

This study does not involve any human or animal testing.

## 6 INFORMED CONSENT

Written informed consent was obtained from all study participants.

## CONFLICT OF INTEREST

The authors declare that they do not have any conflict of interest.
